# Adult Ileocolic Intussusception Secondary to Appendiceal Mucocele: A Report of a Rare Case

**DOI:** 10.7759/cureus.102390

**Published:** 2026-01-27

**Authors:** Ibrahim Y Kutbi, Faris A Zaini, Mohammed A Alnafisah, Hassan A Alherz, Anas E Ahmed

**Affiliations:** 1 College of Medicine, Batterjee Medical College, Jeddah, SAU; 2 College of Medicine, Jeddah University, Jeddah, SAU; 3 College of Medicine, King Saud bin Abdulaziz University for Health Science, Riyadh, SAU; 4 College of Medicine, Sechenov University, Moscow, RUS; 5 Community Medicine, Jazan University, Jazan, SAU

**Keywords:** abdominal pain, adult intussusception, appendiceal mucocele, bowel obstruction, computed tomography, ileocolic intussusception, laparoscopic hemicolectomy, low-grade appendiceal mucinous neoplasm, pseudomyxoma peritonei, surgical management

## Abstract

Adult intussusception is a rare and diagnostically challenging condition, often caused by an underlying pathological lead point such as neoplasms, with presentations that are typically nonspecific and intermittent, including abdominal pain, nausea, vomiting, and altered bowel habits. Among unusual causes, appendiceal mucoceles represent a rare but clinically significant entity that can precipitate intussusception, carrying a risk of serious complications like pseudomyxoma peritonei (PMP) if ruptured. We report the case of a 58-year-old male presenting with right lower quadrant pain, nausea, and intermittent bowel habit changes, in whom imaging revealed ileocolic intussusception with a cystic lesion suggestive of an appendiceal mucocele. The patient underwent laparoscopic-assisted right hemicolectomy, with en bloc resection of the intussuscepted segment and mucocele, avoiding rupture. Histopathology confirmed a low-grade appendiceal mucinous neoplasm without invasion or lymphovascular involvement. The patient recovered uneventfully, and follow-up demonstrated no recurrence or complications. This report highlights the importance of high-resolution imaging in identifying both intussusception and its lead point, the necessity of maintaining suspicion for neoplastic causes in adults, and the critical role of careful surgical management to prevent morbidity and ensure favorable outcomes.

## Introduction

Intussusception in adults is a rare clinical entity, accounting for only 5% of all cases of intussusception and 1-5% of bowel obstructions in the adult population [1,2]. Unlike pediatric cases, which are often idiopathic, adult intussusception is usually secondary to a definable pathological lead point, such as benign or malignant tumors, polyps, or inflammatory lesions [2,3]. The presentation in adults is often nonspecific and chronic, characterized by intermittent abdominal pain, nausea, vomiting, and altered bowel habits, which can lead to delays in diagnosis. Imaging, particularly contrast-enhanced CT, plays a pivotal role in both identifying the intussusception and determining the underlying etiology, which is critical for guiding appropriate management [1-4]. Surgical intervention remains the definitive treatment in adults due to the high likelihood of a pathological lead point and the risk of underlying malignancy [2,3].

Appendiceal mucocele is an uncommon condition resulting from abnormal accumulation of mucin within the appendix, often due to obstruction or neoplastic processes [1,3]. While most cases are asymptomatic and discovered incidentally, appendiceal mucoceles can occasionally act as lead points for intussusception, causing intermittent obstruction or acute abdomen [2,3]. The clinical significance lies in the potential for rupture, leading to pseudomyxoma peritonei (PMP), a serious complication associated with mucinous neoplasms [1,4]. Early recognition through imaging and timely surgical management is essential to prevent morbidity and ensure favorable outcomes [2,3]. Cases of adult intussusception secondary to appendiceal mucocele remain exceedingly rare, with limited reports in the literature, highlighting the importance of awareness among clinicians.

## Case presentation

A 58-year-old male presented to the emergency department with a five-day history of intermittent, crampy abdominal pain predominantly localized to the right lower quadrant, associated with nausea, occasional vomiting, and bloating. He reported a recent change in bowel habits, including episodes of constipation alternating with loose stools, without overt gastrointestinal bleeding. The patient denied any history of fever, weight loss, anorexia, or prior similar episodes. His past medical history was significant only for controlled hypertension, and he had no prior abdominal surgeries. There was no family history of gastrointestinal malignancies. On review of systems, he denied urinary symptoms, jaundice, or systemic manifestations.

On physical examination, the patient appeared mildly distressed due to pain but was hemodynamically stable, with a blood pressure of 128/76 mmHg, heart rate of 92 beats per minute, respiratory rate of 18 breaths per minute, and temperature of 36.8 °C. Abdominal examination revealed a palpable, tender, and slightly mobile mass in the right lower quadrant, without guarding or rebound tenderness. Bowel sounds were hypoactive. There were no signs of peritoneal irritation or hepatosplenomegaly. The remainder of the systemic examination, including cardiovascular, respiratory, and neurological assessments, was unremarkable.

Initial laboratory investigations demonstrated a normal complete blood count, with a white blood cell count of 7.8 × 10⁹/L, hemoglobin 13.6 g/dL, and platelet count 230 × 10⁹/L. Inflammatory markers were mildly elevated, with a C-reactive protein of 12 mg/L. Liver and renal function tests were within normal limits, and serum electrolytes were unremarkable. Tumor markers, including carcinoembryonic antigen (CEA) and carbohydrate antigen 19-9 (CA 19-9), were not elevated.

**Figure 1:**
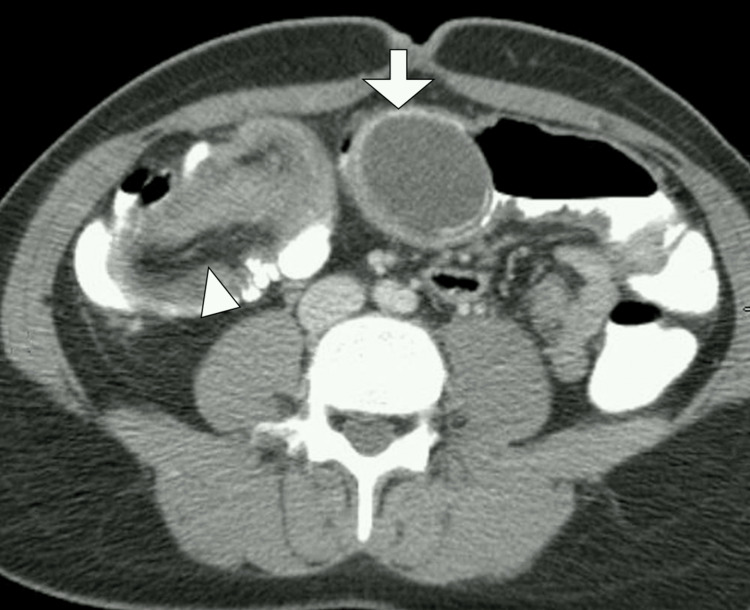
Axial contrast-enhanced CT image of the abdomen and pelvis A distended, fluid-filled appendix consistent with an appendiceal mucocele (arrow) is seen acting as a lead point for ileocolic intussusception (arrowhead) CT: computed tomography

Abdominal CT with intravenous contrast revealed a target-like intussusception in the ileocecal region, extending into the ascending colon. Within the intussuscepted segment, a well-circumscribed, cystic, tubular lesion measuring 4.5 × 3.2 cm was noted at the cecal tip, consistent with a possible appendicular mucocele. The surrounding mesentery appeared mildly edematous, but there was no evidence of free fluid, pneumoperitoneum, or lymphadenopathy. The appendix was distended and demonstrated heterogeneous low attenuation, raising suspicion for a mucinous neoplasm as the lead point of the intussusception (Figure 1).

Given the imaging findings, the differential diagnosis included intussusception secondary to appendiceal mucocele, cecal or appendiceal neoplasm, and, less likely, inflammatory masses such as appendiceal abscess or duplication cyst. The absence of systemic infection, normal inflammatory markers, and the cystic morphology on CT favored a mucinous lesion rather than inflammatory pathology.

The patient underwent an urgent laparoscopic-assisted right hemicolectomy with en bloc resection of the cecum, terminal ileum, and the mucocele-bearing appendix. Intraoperatively, the intussusception was confirmed, and reduction was attempted cautiously to avoid rupture of the cystic appendix. No evidence of peritoneal seeding or gross metastases was observed. The excised specimen included the appendix with an intact mucocele, measuring 5 cm in greatest dimension, without rupture or spillage.

Postoperatively, the patient recovered uneventfully, with gradual advancement of diet and resolution of abdominal pain. He was discharged on postoperative day six in stable condition. At follow-up visits over six months, the patient remained asymptomatic, with no evidence of recurrent intussusception or mucinous dissemination on surveillance imaging. He continues to be monitored periodically with abdominal CT and serum tumor markers to detect any potential recurrence early.

## Discussion

Intussusception in adults represents a distinct clinical entity compared to its pediatric counterpart, both in etiology and management [1-6]. While pediatric intussusception is predominantly idiopathic and often managed non-surgically, adult intussusception is typically secondary to a pathological lead point, with neoplasms accounting for 65-70% of colonic cases and 30-40% of small bowel cases [2,7. The presentation in adults is frequently nonspecific, ranging from intermittent abdominal pain and nausea to subacute bowel obstruction, which often contributes to diagnostic delays [3,5]. Our patient exhibited classical yet subtle features of right lower quadrant abdominal pain, intermittent nausea, and changes in bowel habits, highlighting the insidious nature of adult intussusception and the need for a high index of suspicion. This aligns with previous reports that emphasize the diagnostic challenge in adults, necessitating the use of advanced imaging modalities [1-7].

Contrast-enhanced CT remains the gold standard for diagnosing intussusception in adults, providing both anatomical localization and potential identification of the underlying lead point [3,5,6]. In our case, CT imaging revealed a characteristic “target sign” of ileocolic intussusception, with a well-circumscribed cystic lesion at the cecal tip, suggestive of an appendiceal mucocele [3,6]. Appendiceal mucoceles are rare, occurring in approximately 0.2-0.7% of appendectomies, and can range from simple retention cysts to mucinous neoplasms with malignant potential [1,3,7]. While many mucoceles are asymptomatic, they can occasionally precipitate intussusception when they act as intraluminal masses, as observed in this patient [2,6]. The risk of rupture, leading to PMP, underscores the importance of prompt recognition and careful surgical management. Our case emphasizes that even asymptomatic or incidental mucoceles can present with significant complications, reinforcing the need for thorough preoperative imaging and surgical planning.

Surgical resection remains the cornerstone of management for adult intussusception, both to relieve obstruction and to address the underlying pathology [1,4,6]. En bloc resection without prior reduction is recommended when there is a suspicion of malignancy or when the lead point is located in the colon, to minimize the risk of tumor dissemination [3,5,7]. In this case, a laparoscopic-assisted right hemicolectomy was performed, ensuring complete excision of the appendiceal mucocele and involved ileocecal segment while preserving oncological principles.

This report contributes to the limited literature on adult intussusception secondary to appendiceal mucoceles and underscores several important clinical considerations. First, it highlights the necessity of maintaining clinical suspicion for neoplastic lead points in adults presenting with intermittent abdominal pain or subacute obstruction. Second, it demonstrates the critical role of high-resolution imaging in identifying both the intussusception and its underlying cause, which directly influences surgical planning. Finally, it emphasizes meticulous surgical technique to prevent complications such as mucocele rupture and PMP. Awareness of this rare presentation is essential among gastroenterologists, radiologists, and surgeons to ensure timely diagnosis, safe management, and optimal patient outcomes.

## Conclusions

Adult intussusception secondary to appendiceal mucocele is an exceptionally rare but clinically significant condition that poses diagnostic and therapeutic challenges due to its nonspecific presentation and potential for serious complications such as PMP. Early recognition through high-resolution imaging, particularly contrast-enhanced CT, is essential for identifying both the intussusception and its underlying lead point, guiding timely and appropriate surgical management. Definitive en bloc resection without spillage remains the treatment of choice to ensure complete removal and minimize the risk of recurrence or peritoneal dissemination. This report underscores the importance of maintaining a high index of suspicion for neoplastic causes in adult intussusception and highlights that meticulous preoperative planning and surgical technique are critical to achieving favorable outcomes. The key takeaway is that even rare pathologies like appendiceal mucoceles can present with acute or subacute bowel obstruction, and awareness among clinicians is vital to prevent morbidity and ensure optimal patient care.
